# The correlation of serum sPD-1 and sPD-L1 levels with clinical, pathological characteristics and lymph node metastasis in nonsmall cell lung cancer patients

**DOI:** 10.55730/1300-0144.5407

**Published:** 2022-05-07

**Authors:** Burcu ANCIN, Mesut Melih ÖZERCAN, Yiğit YILMAZ, Serkan UYSAL, Ulaş KUMBASAR, Zeynep SARIBAŞ, Erkan DİKMEN, Rıza DOĞAN, Metin DEMİRCİN

**Affiliations:** 1 Department of Thoracic Surgery, Burdur State Hospital, Burdur, Turkey; 2 Department of Thoracic Surgery, Atatürk Chest Diseases and Thoracic Department of Thoracic Surgery, Surgery Training and Research Hospital, Ankara, Turkey; 3 Department of Thoracic Surgery, Tokat State Hospital, Tokat, Turkey; 4 Department of Thoracic Surgery, Hacettepe University Faculty of Medicine, Ankara, Turkey; 5 Department of Medical Microbiology, Hacettepe University Faculty of Medicine, Ankara, Turkey

**Keywords:** Nonsmall cell lung cancer (NSCLC), soluble programmed cell death protein 1 (sPD-1), soluble programmed cell death ligand 1 (sPD-L1), thoracic surgery

## Abstract

**Background/aim:**

Significant advances have been achieved in immunotherapy for the treatment of lung cancer. It is known that tumor cells and cells in the tumor microenvironment express high amounts of programmed cell death ligand 1 (PD-L1). These PD-L1s interact with programmed cell death protein 1 (PD-1), causing immunosuppression. The aim of our study is to examine the correlation between the serum sPD-1 and sPD-L1 levels and clinicopathological characteristics in patients with nonsmall cell lung cancer. We also compared our results with the healthy population (control group).

**Materials and methods:**

Thirthy-seven nonsmall cell lung cancer (NSCLC) patients who were operated in our clinic were included in our study. The control group included fifteen healthy patients. The sPD-1 and sPD-L1 levels were measured in serum samples by using the ELISA method.

**Results:**

The preoperative sPD-1 and sPD-L1 levels were significantly higher in the study group compared to the control group (44.12 ± 22.25 pg/mL vs. 18.54 ± 6.56 pg/mL; p = 0.001 and 26.15 ± 18.03 pg/mL vs. 10.29 ± 3.08 pg/mL; p = 0.001, respectively). There was a statistically significant decline in serum sPD-1 and sPD-L1 levels at the preoperative and postoperative 1st, 7th, and 30th days following surgical resection (44.12 ± 22.25 pg/mL, 37.86 ± 18.02 pg/mL, 36.33 ± 18.36 pg/mL, 34.14 ± 13.71 pg/mL; p = 0.007 and 26.15 ± 18.03 pg/mL, 20.60 ± 15.50 pg/mL, 18.31 ± 14.04 pg/mL, 13.64 ± 10.60 pg/mL; p = 0.001, respectively).There was a positive correlation between the preoperative and postoperative 30th day serum sPD-1 levels and the tumor size (p = 0.031, r = 0.352; p = 0.024, r = 0.371; respectively). We also found a positive correlation between the preoperative and postoperative 30th day serum sPD-L1 levels and pleural invasion (p = 0.001, p = 0.001; respectively), and the N category (p = 0.015, p = 0.013; respectively).

**Conclusion:**

We think that sPD-1 and sPD-L1 levels may be used as a potential biomarker for lung cancer screening, prediction of the stage, and besides to detect recurrences and/or metastases following resection in NSCLC following validation with multicenter and larger-scale prospective trials.

## 1. Introduction

Cancer is a major health problem with rapidly increasing incidence and mortality worldwide [1]. Numerous genetic and epigenetic changes inherent in most cancer cells provide a large number of tumor-associated antigens that the host immune system can recognize and distinguish them from normal cells [2]. Both the tumor cells and the cells in the tumor microenvironment can express large amounts of programmed cell death-ligand 1 (PD-L1). These PD-L1s interact with programmed cell death protein 1 (PD-1), causing immunosuppression [3]. Therefore, the PD-1/PD-L1 pathway plays an important role in attenuating antitumor immunity in cancers [4]. However, not all cancer types or all cells within a cancer type express PD-L1. It is primarily expressed in nonsmall-cell lung cancer (NSCLC), malignant melanoma, renal cell cancer, stomach cancer, hepatocellular cancer, as well as various leukemias and multiple myeloma-like diseases [5]. This increased expression has also been shown to be associated with poor prognosis and decreased overall survival [6]. Immune checkpoint inhibitors used for immunotherapy have been approved for use in the treatment of many different types of malignancies [7].

There are two distinct expression forms of many costimulatory molecules in immune regulatory pathways, which are membrane-bound form and soluble form. Studies have also shown the presence of soluble forms of PD-1 and PD-L1. Nowadays, there are increasing number of studies showing that serum levels of soluble PD-1 and PD-L1 (sPD-1 and sPD-L1) can be used as biomarkers that might help diagnosis and may be used for predicting prognosis in specific tumors [8,9,10]. This study aimed to investigate the correlation of sPD-1 and sPD-L1 with clinical and pathological characteristics and lymph node metastasis by measuring their serum levels in patients who were operated for early-stage NSCLC and besides to compare preoperative serum sPD-1 and sPD-L1 levels with the post-resection values and with normal population.

## 2. Materials and methods

### 2.1. Patient characteristics

This prospective study included a total of 52 patients. Among them, 37 patients were operated for NSCLC in the Department of Chest Surgery of Hacettepe University Faculty of Medicine Hospital between April 2019 and April 2020 and represented the study group. The control group included fifteen healthy patients who were matched with the study group. There were 30 (81%) male and 7 (19%) female patients with a mean age of 60.41 ± 10.45 years in the study group. The control group included fifteen healthy patients without malignancy. The control group included 10 (66.6%) male and 5 (33.3%) female patients, with a mean age of 39.93 ± 13.25 years. Patients with a diagnosis of secondary cancer were not included in the study. All patients have been informed about the study protocol and all signed a consent form. This prospective research project was approved by the Ethics Committee of Hacettepe University (Decision No: 2019/09-05) and supported by Hacettepe University Scientific Research Projects Coordination Unit. (Project Number: TTU-2019-18220)

### 2.2. Data collection

The preoperative data included age, sex, smoking history, and comorbidities. Postoperative data were the histopathologic type and stage of NSCLC, the presence of lymph node involvement, and the size and invasion characteristics of the tumor in the pathological specimen.

### 2.3. Collection of serum samples

Serum samples were obtained preoperatively, on postoperative days 1, 7, and 30 in the study group and once in the control group. All serum samples were then stored at −80 °C.

### 2.4. Measurement of sPD-1 and sPD-L1 levels using the ELISA method

The sPD-1 and sPD-L1 levels were measured in serum samples by using the enzyme-linked immunosorbent assay (ELISA) method. Human PD-L1 ELISA kit (Invitrogen Thermo Fisher Scientific Inc., USA) with a detection limit of 0.6 pg/mL and human PD-1 ELISA kit (Invitrogen Thermo Fisher Scientific Inc., USA) with a detection limit of 1.14 pg/mL were used in order to measure sPD-L1 and sPD-1 levels, respectively. The tests were performed according to manufacturer’s instructions.

### 2.5. Statistical analysis

Statistical analyses were done using the SPSS version 17.0 software. Histograms and the Kolmogorov–Smirnov test were used to assess the normality distribution of variables. Mean, standard deviation, and median values were used to present descriptive analyses. The Mann–Whitney U test was used for the two-group comparison of nonnormally distributed (nonparametric) variables, while the Kruskal–Wallis test was used for more than two groups. Dunn’s post hoc test was used to test for multiple comparisons between variables for further analysis. After specifying α = 0.01 and 99% power, G-Power 3.0 software package was used and it was determined that a sample size of 11 subjects per group would be required. Spearman’s correlation test was used for the analysis of correlations among quantifiable data. Changes in preoperative, postoperative 1-day, 7-day, and 30-day measurements were analyzed with Friedman’s test. Results with a p-value less than 0.05 were considered statistically significant.

## 3. Results

### 3.1. Comparison of sPD-1 and sPD-L1 levels

**Figure 1:**
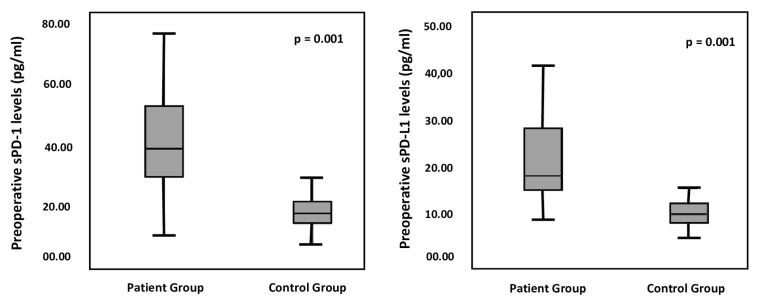
Comparison ofs PD-1 and sPD-L1 levels between the groups(The Mann–Whitney U Test).

**Figure 2:**
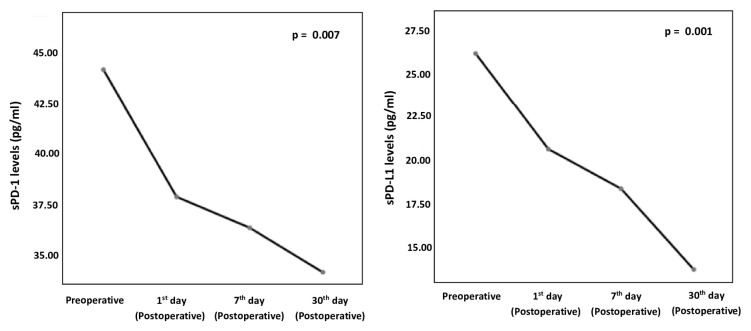
Decline of thesPD-1 and sPD-L1 levels of the study group within the 1st month following surgery (Friedman two-way analysis of variance).

The study group had a statistically significantly higher preoperative sPD-1 levels than the control group (44.12 ± 22.25 pg/mL vs. 18.54 ± 6.56 pg/mL; p = 0.001). Similarly, the study group had a statistically significantly higher preoperative sPD-L1 levels than the control group (26.15 ± 18.03 pg/mL vs. 10.29 ± 3.08 pg/mL; p = 0.001) (Figure 1). The analysis of the alterations in preoperative, postoperative 1st, 7th, and 30th day sPD-1 and sPD-L1 levels of the study group showed a statistically significant decrease in levels of sPD-1 (p = 0.007). Similarly, there was a statistically significant decrease in preoperative and postoperative 1st, 7th, and 30th day sPD-L1 levels. (p = 0.001) (Table 1 and Figure 2).

### 3.2. Tumor size

**Figure 3:**
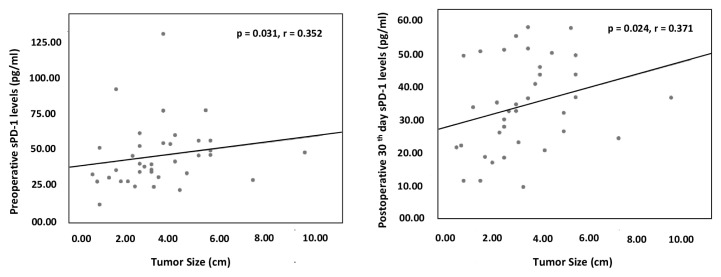
Correlation between the preoperative and postoperative 30th day sPD-1 levels and the tumor size (Spearman’s rho correlation test).

Pathological tumor size (pT) is considered the tumor size. There was a significant positive correlation between the preoperative sPD-1 level and the tumor size (p = 0.031, r = 0.352). There was no significant correlation between the preoperative sPD-L1 level and the tumor size of the patients. The analysis of the postoperative 30th day sPD-1 levels showed a significant positive correlation with tumor size (p = 0.024, r = 0.371). There was no significant correlation between the postoperative 30th day sPD-L1 value and tumor size of the patients (Figure 3).

### 3.3. Histopathological types

The study group was divided into three groups based on their histopathological types: adenocarcinoma, squamous cell carcinoma, and neuroendocrine tumors. There were 17 patients with adenocarcinoma (46%), 12 patients with squamous cell carcinoma (32%), and 8 patients with neuroendocrine tumors (22%). There was no significant difference in terms of preoperative sPD-1 (adenocarcinoma 45.97 ± 20.48 pg/mL, squamous cell carcinoma 49.19 ± 27.51 pg/mL, neuroendocrine tumors 32.58 ± 13.93 pg/mL; p = 0.223) and sPD-L1 levels (adenocarcinoma 31.33 ± 22.58 pg/mL, squamous cell carcinoma 19.29 ± 8.17 pg/mL, neuroendocrine tumors 25.44 ± 15.98 pg/mL; p = 0.184) and postoperative 30th day sPD-1 levels (adenocarcinoma 36.79 ± 16.67pg/mL, squamous cell carcinoma 34.12 ± 7.19pg/mL, neuroendocrine tumors 28.55 ± 13.43 pg/mL; p = 0.450) and sPD-L1 levels (adenocarcinoma 15.40 ± 13.58 pg/mL, squamous cell carcinoma 10.07 ± 4.06 pg/mL, neuroendocrine tumors 15.23 ± 9.94 pg/mL; p = 0.509) among the histopathological types.

### 3.4. T category

The patients were classified according to the T category of the TNM classification based on the data obtained from the postresection pathology results. Of the patients in our study, 17 (49%) had T1 disease, 13 (37%) had T2 disease, and 7 (14%) had T3 disease. There was a statistically significant correlation between the T category and preoperative sPD-1 levels (p = 0.016). According to the post hoc analysis, the mean preoperative sPD-1 level (53.28 pg/mL) of the patients with a T2 tumor was statistically significantly higher than the mean preoperative sPD-1 value (33.07 pg/mL) of the patients with a T1 tumor. The analysis of the correlation between the T categories and postoperative 30th day sPD-1 levels of the patients revealed a statistically significant difference (p = 0.023). Post hoc analysis showed that the mean postoperative 1st month sPD-1 level of the patients with a T3 tumor (43.51 pg/mL) was statistically higher than the mean postoperative 1st month sPD-1 level of the patients with a T1 tumor (27.78 pg/mL). There was no correlation between the T category and sPD-L1 levels of the patients.

### 3.5. N category

The patients were classified according to the N (lymph node) category based on the data obtained from the postsurgical resection pathology results. There were 31 patients (84%) with N0 disease and 6 patients (16%) with N1 disease. There was a positive correlation between the N category and preoperative sPD-L1 levels. Preoperative sPD-L1 levels significantly increased as the N category increased (p = 0.015). However, there was no correlation between the N category and the preoperative sPD-1 levels of the patients. The postoperative 30th day sPD-L1 levels significantly rose in higher N categories (p = 0.013), whereas there was no correlation between the N categories and postoperative 30th day sPD-1 levels.

### 3.6. Pleural invasion

The patients with pleural invasion had a significantly higher presurgical sPD-L1 levels than those of without invasion (p = 0.001). Similarly, the patients with pleural invasion had significantly higher postoperative 30th day sPD-L1 levels than those of without invasion (p = 0.001). However, no statistically significant correlation was detected between sPD-1 levels and pleural invasion (Table 2).

## 4. Discussion

Lung cancer is the most frequently diagnosed cancer type with the highest mortality in the world [11]. Numerous genetic and epigenetic alterations occur in all cancers, leading to the production of a large number of antigens in tumor cells. Thus, cancer cells are recognized by the immune system and distinguished from normal cells [2,11]. Remarkable advances have been achieved in cancer immunotherapy with the discovery of various antigens used by the immune system in recent years [7]. The PD-1/PD-L1 pathway is one of the most useful immune checkpoints in the treatment of cancer patients [4]. PD-L1 is also present in the cytoplasm and plasma membrane of nonsmall cell lung cancer cells [5]. These PD-L1s interact with PD-1, causing immunosuppression [3]. Increased PD-L1 expression has also been shown to be associated with poor prognosis and decreased overall survival [6]. There are two distinct expression forms of a large number of costimulatory molecules in immune regulatory pathways, which are membrane-bound form and soluble form. The measured blood levels of soluble PD-1 and PD-L1 are useful in predicting the clinicopathological characteristics, treatment response, and survival outcomes of cancer patients [9].

In their study, Okumo et al. showed significantly higher sPD-L1 levels in advanced stage NSCLC patients compared to the healthy control group [10]. In another study which compared sPD-1 levels of 66 patients with triple-negative breast cancer and 59 healthy individuals, higher sPD-1 levels were detected in the study arm [12]. In line with the mentioned studies, our study also showed significantly higher preoperative sPD-L1 and sPD-1 levels in the study group.

Surgery is still the first-line treatment modality for patients with early-stage NSCLC. However, studies have shown that 30%–50% of patients develop recurrence after the surgery. Thus, postoperative follow-up of these patients is of paramount importance [13]. We measured both preoperative sPD-1 and sPD-L1 levels in patients who underwent surgical resection and compared these levels with the postresection values on the 1st, 7t, and 30th days. We showed a statistically significant decrease in both sPD-1 and sPD-L1 levels in the postoperative period. Similarly, in a recent study conducted in 24 patients with hepatitis B virus-associated hepatocellular cancer and who underwent hepatobiliary surgery, researchers demonstrated a significant decrease in sPD-1 levels postoperatively [14]. In light of these studies, we postulate that sPD-1 and sPD-L1 levels increase in proportion to tumor burden and can potentially be used as a marker in the postoperative follow-up of NSCLC.

He et al. investigated the correlation between tumor size and sPD-1 and sPD-L1 levels in 88 NSCLC patients but were unable to find any correlation [15]. However, Frigola et al. reported that sPD-L1 levels were associated with tumor size in patients with renal cell cancer [16]. Our study showed a positive correlation between the tumor size and the sPD-1 levels in the preoperative and postoperative 1st month. However, there was no significant difference between sPD-L1 levels and tumor size measured in the same period.

It is known that there are various immunosuppression mechanisms mediated by the PD-1/PD-L1 pathway in the tumor microenvironment and that PD-L1 is highly expressed by tumor cells and other cells in the tumor microenvironment. Similarly, PD-1 is expressed by cells in the tumor microenvironment and its soluble form is released into the circulation [17]. In light of these data, we believe that sPD-L1 and sPD-1 levels are increasing parallel to the increase in tumor burden, as also observed in other tumor markers. We think that the discrepancies between the studies may be due to the small number of patients, the heterogeneity of the patient groups, and the newly used ELISA methods for the determination of soluble forms.

Okuma et al. showed no correlation between the sPD-L1 levels and NSCLC histopathologic subtypes [18]. Consistently, our study also did not show any correlation between the histopathological types and preoperative and postoperative 30th day sPD-1 and sPD-L1 levels.

Cheng et al. compared sPD-L1 levels in various stages of NSCLC and showed that sPD-L1 levels are higher in stage 3 and 4 compared to early stages [19]. However, Okuma et al. showed no correlation between the stage of cancer and sPD-L1 levels in patients with advanced NSCLC [10]. There is no study in the literature demonstrating the correlation between the T category of the TNM staging system and the levels of sPD-1 and sPD-L1. We think that it would be more useful to make a comparison with the T category instead of the stage since our study was conducted solely on early-stage patients who underwent surgical treatment. We showed a significant correlation between the T category and preoperative sPD-1 levels. The post hoc analysis revealed significantly higher sPD-1 levels in T2 tumors compared to T1 tumors. Also, the analysis of the correlation between the T category and postoperative 30th day sPD-1 levels of the patients showed a statistically significant difference. However, we did not find any correlation between the T category and sPD-L1 levels. Most of the measured levels of sPD-L1 are caused by PD-L1 found on the surface of tumor cells. Likewise, PD-1 is expressed by cells in the tumor microenvironment and its soluble form is released into the circulation [17]. In view of these data, we predict a rise of sPD-L1 and sPD-1 levels in correlation with the increase in the T category. Nevertheless, we could only observe this rise only for certain T categories. We believe that this is due to our insufficient sample size.

To our knowledge, there is no study in the literature which shows the correlation between pleural invasion and sPD-1 and sPD-L1 levels. In a study of 141 patients with hepatitis B virus-associated hepatocellular cancer, a correlation was found between circulating PD-L1 levels and vascular invasion [20]. Tominaga et al. also showed a significant association between high sPD-L1 levels and lymphovascular invasion in patients with locally advanced rectal cancer [21]. In our study, we observed higher preoperative sPD-L1 levels in patients with pleural invasion compared with those without invasion. In addition, postoperative 30th day sPD-L1 levels were significantly higher in patients with pleural invasion. However, there was no significant correlation between pleural invasion and sPD-1 levels. The soluble molecular form of costimulatory molecules is generated by proteolytic degradation of the membrane-bound form of proteins. Therefore, higher serum concentrations of sPD-L1 should be expected in patients with pleural invasion, which is also supported with the results of our study.

The mechanisms by which malignant tumors separate from their primary sites and metastasize to regional lymph nodes are complex. Following tumor growth, lymphangiogenic cytokines are released, leading to the development of new lymphatic vessels towards the tumor. Tumor cells migrate to lymph nodes through these lymphatic capillaries. Angiogenesis around the tumor occurs similarly. Cytokines that play a role in angiogenesis and lymphangiogenesis are similar [22]. Zheng et al. found a significant correlation between the expression level of circulating PD-L1 and lymph node metastasis in patients with advanced stomach cancer [23]. Our analysis also showed that the preoperative sPD-L1 levels significantly increased as the N category increased. However, there was no correlation between the N categories and preoperative sPD-1 levels of the patients. Similarly, the postoperative 30th day sPD-L1 levels measured significantly higher as the N category increased, while the sPD-1 levels showed no significant difference in the same period. Therefore, a positive correlation between the levels of circulating PD-L1 and the N category is expected.

Our study had limitations that should be addressed. First of all, this study was conducted at a single center in Turkey, which may limit generalizability of the results to larger populations. Secondly, the sample size is relatively small. Thirdly, the follow-up period was only 1 month; therefore, the role of levels of circulating PD-1 and PD-L1 as a tumor marker was difficult to analyze.

## 5. Conclusion

In conclusion, our study demonstrated that sPD-1 and sPD-L1 levels can be used as a potential biomarker for lung cancer screening, prediction of the stage, and besides to detect recurrences and/or metastases following resection in NSCLC. However, our data deserve further verification with multicenter and larger-scale prospective trials.

## Figures and Tables

**Table 1:** sPD-1 and sPD-L1 levels of the study group.

		**n**	Mean	Median	SD	p	Post hoc
sPD-1 levels (pg/mL)	Preoperative	37	44.12	38.79	22.25	0.007*	1–2 1–3 1–4
	Postoperative 1st day	37	37.86	32.65	18.02		
	Postoperative 7th day	37	36.33	31.07	18.36		
	Postoperative 30th day	37	34.14	33.64	13.71		
sPD-L1 levels(pg/mL)	Preoperative	37	26.15	18.31	18.03	0.001*	1–2 1–3 1–4 2–4 3–4
	Postoperative 1st day	37	20.60	15.65	15.50		
	Postoperative 7th day	37	18.31	12.91	14.05		
	Postoperative 30th day	37	13.64	9.48	10.60		

Friedman two-way analysis of variance

SD: standard deviation

* statistically significant

**Table 2:** Pre- and postoperative sPD-1 and sPD-L1 levels among subgroups.

	Median	SD	p-value	Median	SD	p-value
	Preoperative sPD-1 levels (pg/mL)			Preoperative sPD-L1 levels (pg/mL)		
**T1 (**n = **17)**	33.07	±10.03	0.016 b* (T1–T2)^c^	17.60	±8.34	0.291 b
**T2 (**n = **13)**	53.28	±29.77		18.27	±22.36	
**T3 (**n = **7)**	46.78	±16.02		28.30	±23.19	
**N0 (**n = **31)**	38.79	±21.71	0.510^a^	17.60	±14.85	0.015^a^*
**N1 (n = 6)**	46.74	±26.75		39.99	±22.63	
	Postoperative 30th day sPD-1 levels (pg/mL)			Postoperative 30th day sPD-L1 levels (pg/mL)		
**T1 (**n = **17)**	27.78	±11.92	0.023 b* (T1–T3) c	9.37	±7.22	0.424 b
**T2 (**n = **13)**	40.65	±14.40		9.37	±14.20	
**T3 (**n = **7)**	43.51	±11.03		14.11	±10.32	
**N0 (**n = **31)**	32.43	±12.86	0.122^a^	9.37	±7.21	0.013^a^*
**N1 (**n = **6)**	47.87	±16.68		26.62	±16.26	
	Preoperative sPD-1 levels (pg/mL)			Preoperative sPD-L1 levels (pg/mL)		
**Pleural** invasion -	38.41	±20.41	0.088^a^	16.76	±12.62	0.001^a^*
**Pleural invasion +**	68.21	±26.45		59.73	±17.27	
	Postoperative 30th day sPD-1 levels (pg/mL)			Postoperative 30th day sPD-L1 levels (pg/mL)		
**Pleural** invasion -	32.49	±12.42	0.185^a^	9.15	±5.83	0.001^a^*
**Pleural** invasion +	50.71	±19.13		29.26	±15.74	

a The Mann–Whitney U test

b The Kruskal–Wallis test

c Dunn’s post hoc test

SD: Standard deviation,

* statistically significant
